# The Electrophysiological Underpinnings of Processing Gender Stereotypes in Language

**DOI:** 10.1371/journal.pone.0048712

**Published:** 2012-12-03

**Authors:** Anna Siyanova-Chanturia, Francesca Pesciarelli, Cristina Cacciari

**Affiliations:** 1 Department of Biomedical Sciences, University of Modena and Reggio Emilia, Modena, Italy; 2 School of Linguistics and Applied Language Studies, Victoria University of Wellington, Wellington, New Zealand; University of Leicester, United Kingdom

## Abstract

Despite the widely documented influence of gender stereotypes on social behaviour, little is known about the electrophysiological substrates engaged in the processing of such information when conveyed by language. Using event-related brain potentials (ERPs), we examined the brain response to third-person pronouns (*lei* “she” and *lui* “he”) that were implicitly primed by definitional (*passeggera*_FEM_ “passenger”, *pensionato*_MASC_ “pensioner”), or stereotypical antecedents (*insegnante* “teacher”, *conducente* “driver”). An N400-like effect on the pronoun emerged when it was preceded by a definitionally incongruent prime (*passeggera*_FEM_ – *lui*; *pensionato*_MASC_ – *lei*), and a stereotypically incongruent prime for masculine pronouns only (*insegnante – lui*). In addition, a P300-like effect was found when the pronoun was preceded by definitionally incongruent primes. However, this effect was observed for female, but not male participants. Overall, these results provide further evidence for on-line effects of stereotypical gender in language comprehension. Importantly, our results also suggest a gender stereotype asymmetry in that male and female stereotypes affected the processing of pronouns differently.

## Introduction

The ways in which people acquire, store, represent and process social stereotypes is central to the domain of social cognition. Stereotypes represent a form of social knowledge that is linked to actions, attitudes, rules and other forms of knowledge and behavioural representation [1]. These representations constitute a precompiled semantic knowledge about the world, which may be automatically activated whenever one refers to another person with a word conveying a stereotype (e.g., a role noun [2]). Social psychologists [2], [3] showed that specific gender-oriented stereotypes (i.e., gender-oriented beliefs about the attributes of social groups) are associated with many English words, especially role nouns, and are automatically activated whenever such stereotyped role nouns are encountered in discourse. These effects have been replicated in psycholinguistic studies on Spanish, Italian, and German [4]–[13].

Overall, psycholinguistic studies showed that words that convey exemplars that are incongruent with a gender stereotype (e.g., male nurse/female doctor) are processed slower than congruent ones (e.g., female nurse/male doctor). However, relatively little is known about the electrophysiological correlates engaged in processing gender stereotypes [14]. As noted by some researchers [15], behavioural measures represent the outcome of a set of cognitive processes, but are not, themselves, direct measures of these processes. Event-related brain potentials (ERPs), on the other hand, can more directly measure the responses that reflect cognitive and affective processes of interest to social cognition [15].

### ERPs in gender stereotype processing

Existing research suggests a strong relationship between ERPs and the processing of anomalous social information [16]. The few ERP studies on gender stereotypes in language showed that a mismatch between an antecedent conveying a gender stereotype and a referent that follows it (e.g., *nurse – himself*, *pilots – these women*, *aggressive - women*) can be indexed by two ERP components, the N400 and the P600. The N400, a negative-going deflection peaking around 400 ms after stimulus onset, is modulated by several factors, such as violation of semantic information and world knowledge [17]–[20]. In addition, the N400 reflects the difficulty of accessing information from semantic memory [21]. White et al. [22] presented participants with a gender category (*men/women*) followed by a word conveying a characteristic stereotypically associated with males or females (e.g., *aggressive/nurturing*). Participants were required to explicitly judge whether or not the two words matched according to their gender stereotypical beliefs. Stereotypically incongruent word pairs (*men/nurturing*, *women/aggressive*) elicited larger N400s than congruent ones. In Irmen et al. [23], participants read statements concerning specific occupations (e.g., *florists*, *pilots*) followed by masculine, feminine, or neutral gender noun phrases (*these men/women/people*). In the N400 time window, the authors observed a greater negativity across all anaphors (masculine, feminine, neutral) following a typically male than female antecedent. Why does the violation of a gender stereotype elicit an N400 effect? It appears that gender stereotypes convey semantic-pragmatic information whose violation elicits a brain response similar to that elicited by pragmatically incongruent words. The perceived anomaly may result from an evaluation of the pragmatic plausibility of a male playing a stereotypically female role, or, conversely, of a female playing a stereotypically male role [14].

The second component elicited by gender stereotype violations is the P600, a slow positive shift emerging in a time window around 500–900 ms after stimulus onset. Traditionally, the P600 is considered to index syntactic processing difficulties [24]–[25]. Recent studies, however, observed this component also in the context of non-syntactic linguistic manipulations [26]–[28]. In a study that investigated the processing of reflexive pronouns, Osterhout, Bersick, and McLaughlin [14] obtained P600 effects when the stereotypical gender of an antecedent role noun disagreed with the gender of a reflexive pronoun that followed it (*doctor – herself*). Similarly, the P600 was found when the definitional gender of an antecedent role noun and the gender of a reflexive pronoun disagreed (*woman – herself*). The effect of stereotypical gender, however, was smaller than that of definitional gender. The P600 effect observed in response to stereotype violations may reflect processes involving re-integration of semantic meaning and stereotypical beliefs. Participants initially assigned the preferred (i.e., stereotypical) gender feature to stereotypically male or female nouns, but were subsequently forced to assign the less preferred gender feature upon encountering a reflexive pronoun inconsistent with the stereotype. In Irmen et al.'s study [23], the authors also found an interaction between antecedent typicality and anaphor gender in the P600 window, with more positive brainwaves when grammatically feminine anaphors were incongruent with the antecedent gender typicality than when they were congruent. Finally, in Lattner and Friederici [29], participants heard sentences that contained stereotypically male or female self-referent statements (e.g., *I like to wear skirts*) spoken by male or female voices. The integration of speaker voice and stereotypical knowledge occurred rather late and was reflected in a P600 effect, elicited when the sentence was voiced by a stereotypically incongruent speaker (e.g., *I like to wear skirts* spoken by a male). However, these results were not supported by those of Van Berkum et al. [30]. In Van Berkum et al. [30], participants listened to sentences whose content mismatched inferences based on the identity of the speaker (e.g., *If only I looked like Britney Spears* in a male voice, or *I have a large tattoo on my back* spoken with an upper-class accent), or contained semantic anomalies (e.g., *Dutch trains are sour and blue* again voiced by male or female speakers). The speaker's identity was taken into account as early as 200–300 ms after the beginning of the critical word, and elicited the same type of brain response as semantic anomalies – the N400.

Overall, these few studies suggest that where stereotypical role nouns are embedded in sentential contexts, their violation is more likely to elicit P600 effects [14], [23], [29]. In contrast, where single role nouns are presented in semantic/associative priming paradigms, violations of gender stereotypes are typically accompanied by N400 effects [22]. This different pattern of results for word pairs versus sentences has an interesting parallel in the literature on grammatical agreement violations. Barber and Carreiras [31] showed that grammatical gender agreement violations in Spanish noun-adjective and article-noun pairs elicited the N400 [as well as a left anterior negativity (LAN) for the latter]. However, agreement violations with the same word pairs inserted in sentences resulted in LAN-P600 effects. These different patterns were interpreted as reflecting different processes: integration of lexical features for word pairs (indexed by the N400) and syntactic parsing and construction of a syntactic structure for sentences (indexed by the LAN-P600 complex).

Grammatical gender processing has been investigated in many more ERP studies than stereotypical gender presumably because of the relevant role of grammatical gender in processing agreement relationships in sentence comprehension (for an overview, see [32]). Typically, syntactic agreement mismatch elicits a biphasic electrophysiological pattern (LAN-P600). However, agreement relationships may also involve non-syntactic high-level information, whose violation is typically indexed by the N400. For instance, many studies on anaphor processing showed that gender violations elicit N400 effects, sometimes in addition to P600 effects. Specifically, an N400-P600 complex was obtained when noun antecedents characterized by grammatical gender mismatched the gender of the pronouns [33]–[35].

### Objectives of the present study

The present study was designed to investigate the electrophysiological correlates of the on-line processing of definitional and stereotypical gender conveyed by single words. We examined the electrophysiological response to Italian pronouns when they were implicitly primed by definitional and stereotypical antecedent words. To this aim, we adapted the gender-priming paradigm of Banaji and Hardin [2]: participants were visually presented with a prime word characterized by definitional (e.g., *passeggera*_FEM_ “passenger”, *pensionato*_MASC_ “pensioner”), or stereotypical gender (e.g., *insegnante* “teacher”, *conducente* “driver”). A prime was followed by a grammatically masculine or feminine personal pronoun (e.g., *lui* “he” and *lei* “she”). Participants were asked to decide whether the pronoun was grammatically masculine or feminine. Behavioural evidence obtained using this paradigm [2], [4] showed that stereotypical word primes triggered activation of the gender stereotypes within 500–600 milliseconds, similar to definitional primes. If, in the present study, we observe P600 effects for both types of gender violation, thus replicating Osterhout, Bersick, and McLaughlin [14], this may index increased processing demands arising from higher-level conceptual complexity [36]–[38]. In addition, the P600 may signal a more general activation associated with anomaly or incongruence detection [39]–[40]. However, stereotypes may also be conceived as a form of precompiled world knowledge [41], whose violation is typically accompanied by the N400 [17]. In addition, the N400 is known to be modulated by the ease of retrieval of conceptual knowledge from long-term memory [31], [42], [43]. Thus, violations of stereotypical gender may modulate the N400. Violations of definitional gender may elicit P600 effects [14], [44] and/or an N400/P600 complex [33]–[35].

Barber and Carreiras [45] reported a posterior P300 effect on grammatical gender (and number) agreement violations in noun-adjective pairs. This effect was interpreted as indexing a response-related binary decision (the categorization of a word pair as wrong). Since the present study employs word pairs and a binary decision (classifying a target pronoun as masculine or feminine), we may also find an N400 effect followed by a P300 effect. The P300s form a family of functionally distinct components related to the subjective probability of the eliciting event [46], [47]. This family includes a more anterior P300a (*novelty* P300), typically elicited by unexpected events, and a more posterior P300b elicited by infrequent task-relevant stimuli with a latency varying as a function of the time necessary to categorize a rare event [47]–[49]. In their study on gender stereotypes, Osterhout, Bersick, and McLaughlin [14] discussed the possibility that the positive shift they observed might be a member of the P300 family instead of a P600. Specifically, the different amplitudes of the violations of stereotypical versus definitional gender may reflect the subjective probability of encountering a woman or a man in various occupational roles or states. However several factors (e.g., lack of covariance between positive shift and event probability, distinct morphologies, time courses, scalp distributions, and differential sensitivities to changes in task and probability) led Osterhout, Bersick, and McLaughlin [14] to discard the P300 account of the observed positive shift.

Lastly, because participants may vary in the strength of their personally held stereotypical beliefs and associations, we included a series of explicit post-EEG measures (see Method).

## Methods

### Participants

Twenty-seven University of Modena and Reggio Emilia students [14 females, mean age 24 (range 19–34), *SD* 4.7] participated in the experiment for course credit or payment (€15). All were right-handed as assessed by the Edinburgh handedness inventory [50] and had normal or corrected-to-normal vision. None of the participants reported a history of prior neurological disorder. All participants were informed of their rights and gave written informed consent for participation in the study, according to the Declaration of Helsinki. The research was carried out fulfilling ethical requirements in accordance with the standard procedures of the University of Modena and Reggio Emilia.

### Materials

#### Norming phase

260 words (nouns, past participles and adjectives) specifying occupations, roles and individual characteristics were included in written questionnaires (each containing 130 words) that were presented to 40 students (20 females) not further involved in the experiment. Participants were asked to rate the extent to which each word was associated with men or women, or both, using a seven-point Likert scale. The labels of the scale poles (1 – only men, 4 – both, 7 – only women) were reversed for half of the participants. The final rating assigned to each word was calculated by combining the ratings obtained with both directions of the rating scale. The 60 words selected as experimental primes in the stereotypical gender condition received high ratings of male-oriented or female-oriented stereotypicality (Table 1). The strength of male- and female-oriented stereotypes did not differ (*p*>.05). When presented in isolation, these words are bi-gender since they do not convey any information about the biological gender of the referent. To avoid any interference of the gender-to-ending consistency typical of Italian, all the stimuli in the stereotypical condition ended in –e (46 words out of 60), or in a consonant (14 out of 60). Thirty primes had an associated male stereotype (e.g., *conducente* “driver”), and 30 had an associated female stereotype (e.g., *insegnante* “teacher”) (see Table S1).

**Table 1:** Mean log frequency, length, stereotypicality, and valence for stereotypical and definitional gender stimuli.

	Stereotypical		Definitional		
	feminine	masculine	feminine	masculine	
**Log frequency**	2.8 (0–4.6)^1^	2.9 (1.1–4.8)	2.9 (1.5–4.4)	2.9 (0.8–4.7)	*p>.05*
	*0.9*	*0.7*	*0.8*	*0.7*	
**Length**	8.0 (4–10)	7.8 (5–10)	8.0 (4–10)	7.8 (5–10)	*p>.05*
	*1.8*	*1.4*	*1.8*	*1.4*	
**Stereotypicality**	5.3 (4.6–6.5)	2.4 (1.7–3.1)	4.0 (3.8–4.2)	4.0 (3.8–4.2)	
**(raw ratings)**	*0.6*	*0.4*	*0.1*	*0.1*	
**Stereotypicality**	2.7 (1.5–3.4)*	2.4 (1.7–3.1)*	4.0 (3.8–4.2)**	4.0 (3.8–4.2)**	*p>.05
**(normalized)** 2	*0.6*	*0.4*	*0.1*	*0.1*	**p>.05
**Valence**	4.5 (2.3–6.1)	4.2 (1.7–5.6)	4.2 (1.7–6.3)	4.3 (2.2–5.6)	*p>.05*
	*1.1*	*1.0*	*1.2*	*1.1*	

1 Min and max ratings are indicated in parenthesis. Standard Deviation is indicated in italics below the means. T-tests were performed on the means; no statistically significant differences were found in any of the comparisons, suggesting that the stimuli were closely matched for the above properties.

2 The final rating assigned to each word was calculated by combining the ratings obtained with both directions of the rating scale (see Method).

In order to select 60 items without any stereotypical bias for the definitional condition, another questionnaire with 138 words with definitional gender was given to 40 students (20 females), not further involved in the experiment. We selected 60 words whose definitional gender was morphologically marked in the final vowel. Thirty words ended in –a (feminine definitional condition), and 30 in –o (masculine definitional condition) (Table 1). Some of the target words selected were nominalized past and present participles (20 out of 120), or nominalized adjectives (14 out of 120). In Italian, such forms are commonly used as nouns (e.g., *pensionato* “pensioner”, *conducente* “driver”, *sexy* “sexy”, [51]). Definitional primes had no stereotypical associations. The same word appeared either with a feminine or masculine inflection, but not both (e.g., *pensionato* or *pensionata*) (see Table S1).

Unlike Banaji and Hardin [2] we did not use words that have distinct forms for male and female individuals (e.g., *dottore*_MASC_ – *dottoressa*_FEM_). The four types of stimuli (stereotypically male or female; definitionally male or female) were matched for written frequency (*La Repubblica* corpus, [52]) and length (number of characters) (Table 1). Finally, 30 filler role nouns ending in –e, without any associated gender stereotype (e.g., *conoscente* “acquaintance”), were included to prevent participants from noticing the presence of the gender-stereotyped words.

Because the valence of our experimental stimuli varied, in another questionnaire, we asked 40 students (20 females), not further involved in the experiment, to rate the valence of our experimental words using a seven-point Likert scale (half of the respondents saw: 1 – negative, 4 – neutral, 7 – positive; the other half saw a reversed scale). The mean valence ratings of the words in the four conditions did not significantly differ (*p*>.05) (Table 1).

The targets were third-person masculine and feminine pronouns (*lui* “he” and *lei* “she”) that have a comparable written frequency (the log frequency, calculated using *La Repubblica* corpus, is 5.4 for *lui* and 5.1 for *lei*). Each prime was paired with a pronoun so as to form congruent pairings (definitional: *pensionato – lui*, *passeggera – lei*; stereotypical: *conducente – lui, insegnante – lei*), and incongruent pairings (definitional: *pensionato – lei, passeggera – lui*; stereotypical: *conducente – lei, insegnante - lui*).

### Procedure

Participants were seated comfortably in a darkened sound-attenuated room. Stimuli were presented in light grey upper case letters (Courier font, size 13) against a black background on a high-resolution computer that was positioned at eye level circa 70 cm in front of the participant.

The procedure was modelled after that of Banaji and Hardin's [2] study. A fixation point (+) appeared in the middle of the screen and stayed there until participants pressed a button to start a trial. Then a blank screen was displayed for 500 ms. The prime was displayed on the screen for 300 ms followed by a blank screen for 200 ms. Finally, the target pronoun (*LEI* or *LUI*) appeared and remained on the screen until a response was made. Participants were instructed to press the response button marked with M if the pronoun was masculine, and F if it was feminine. Half of the participants had M on the left and F on the right, while the other half had the position of the response buttons reversed. Each response was followed by a 1000 ms blank screen.

The instructions were presented in written form on the computer screen before the experiment started. Participants were asked to decide as quickly as possible whether the pronoun was grammatically masculine or feminine ignoring the prime. The same prime was followed by a feminine target pronoun in one block and then by a masculine target pronoun in another block. Before starting the experiment, participants took part in a short training session with 20 prime-target pairs (half of the primes were masculine and half feminine) formed by stimuli different from those used in the experimental session. The training session was followed by three experimental blocks of 100 trials each containing an equal number of definitionally and stereotypically male and female primes, and an equal number of feminine and masculine pronouns, as well as an equal number of fillers. The trial presentation sequence was randomized.

In order to measure individual stereotypical gender attitudes, following the EEG registration, participants completed the *Bem Sex Role Inventory* (BSRI) [53] and the *Ambivalent Sexism Inventory* (ASI) [54]. The ASI has two scales that measure two correlated components of sexism representing opposite evaluative orientations towards women: sexist antipathy – *Hostile Sexism* (HS), and a subjectively positive orientation towards women – *Benevolent Sexism* (BS).

### Electroencephalograph (EEG) recording and analysis

EEG was amplified and recorded with the BioSemi Active-Two System from 30 active electrodes placed on the scalp (AF3, AF4, F3, F4, F7, F8, FC1, FC2, FC5, FC6, C3, C4, T7, T8, CP1, CP2, CP5, CP6, P3, P4, P7, P8, O1, O2, PO3, PO4, Fz, Cz, Pz, Oz). In addition, four electrodes were placed around the eyes for eye-movement monitoring (two at the external ocular canthi and two below the eyes) and two electrodes were placed over the left and right mastoids. Two additional electrodes were placed close to Cz, the Common Mode Sense [CMS] active electrode and the Driven Right Leg [DRL] passive electrode, that were used to form the feedback loop that drives the average potential of the participant as close as possible to the AD-box reference potential [55]. EEG and EOG signals were amplified and digitized continuously with a sampling rate of 512 Hz. EEG signals were referenced off-line to the average activity of the two mastoids and then analyzed using Brain-Vision Analyzer.

After a band-pass filter (0.01–80 Hz band pass), 1200-msec epochs containing the ERP elicited by the target pronoun were extracted, starting with 200 ms prior to the onset of the pronoun. Data with excessive blinks were adaptively corrected using ICA. Segments including artefacts (such as excessive muscle activity) were eliminated off-line before data averaging. Two participants were excluded from all the analyses due to the high number of rejected epochs (>25%). The lost data (due to artefacts) of the remaining 25 participants were equal to 5.2%. A 200 ms pre-stimulus baseline was used in all analyses.

## Results

### Behavioural results

**Figure 1:**
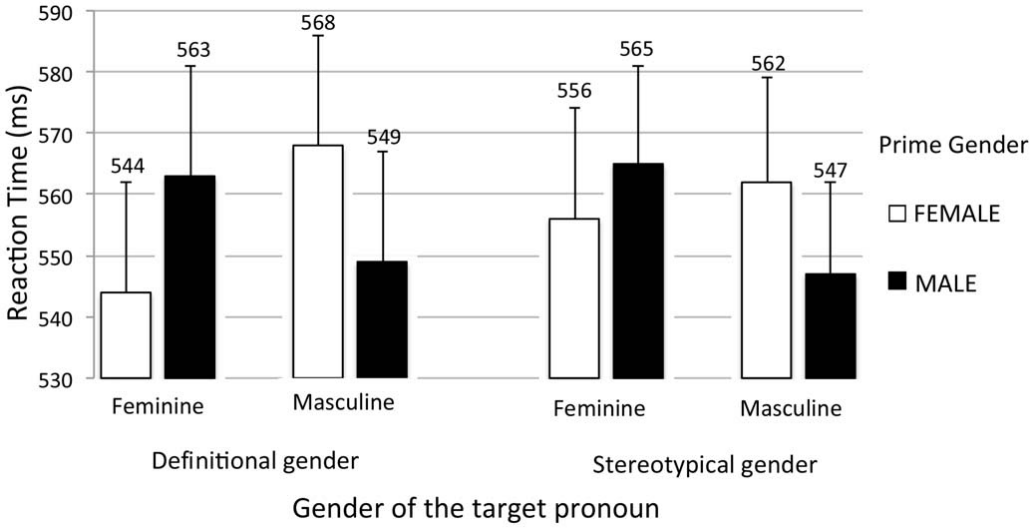
Mean reaction times to judge whether the target pronoun was feminine or masculine.

Individual reaction times (RTs) exceeding ±2 SD were eliminated (0.74%). The mean error rate was 2.7%. Figure 1 shows the mean RTs to decide the gender of the target pronouns in the definitional and stereotypical conditions. The data were analyzed in an omnibus 2 (Prime Type: definitional, stereotypical)×2 (Prime Gender: female, male)×2 (Target Gender: feminine, masculine) ANOVA. In addition, separate ANOVAs were run on definitional and stereotypical gender primes. In the *t*-tests reported below, we estimated the effect size by means of Cohen's *d* [56], [57].

As the effects under investigation might be influenced by participant gender, a between subject effect of participant gender was included in all ANOVAs conducted. Because the main effects of Participant Gender, Prime Gender, and Target Gender, as such, are not central to the question under investigation, they are not reported. Below, we discuss only those interactions that are of interest to our study.

The omnibus ANOVA revealed a significant Prime Gender×Target Gender interaction [F(1, 23) = 20.7, *p*<.001, **η**_p_^2^ = .48]. Separate ANOVAs showed a gender priming effect for definitional [*F*(1, 23) = 16.9, *p*<.001, **η**_p_^2^ = .42], and stereotypical primes [*F*(1, 23) = 13.5, *p*<.005, **η**_p_^2^ = .37].

The gender decision on the pronoun was influenced by the gender congruency of the prime for both definitional and stereotypical primes. Participants were faster to judge masculine pronouns when preceded by male than female definitional primes [*M* = 549, *SD* = 92; *M* = 568, *SD* = 90, respectively; *t* (24) = 5.5, *p*<.001, *d* = .2], and by male than female stereotypical primes [*M* = 547, *SD* = 74; *M* = 562, *SD* = 87, respectively; *t*(24) = 2.7, *p* = .01, *d* = .2]. Likewise, participants were faster to judge feminine pronouns when preceded by female than male definitional primes [*M* = 544, *SD* = 90; *M* = 563, *SD* = 89, respectively; *t*(24) = 2.2, *p* = .04, *d* = .2], and by female than male stereotypical primes [*M* = 556, *SD* = 89; *M* = 565, *SD* = 81, respectively; *t*(24) = 2.3, *p* = .03, *d* = .1].

### ERP results

**Figure 2:**
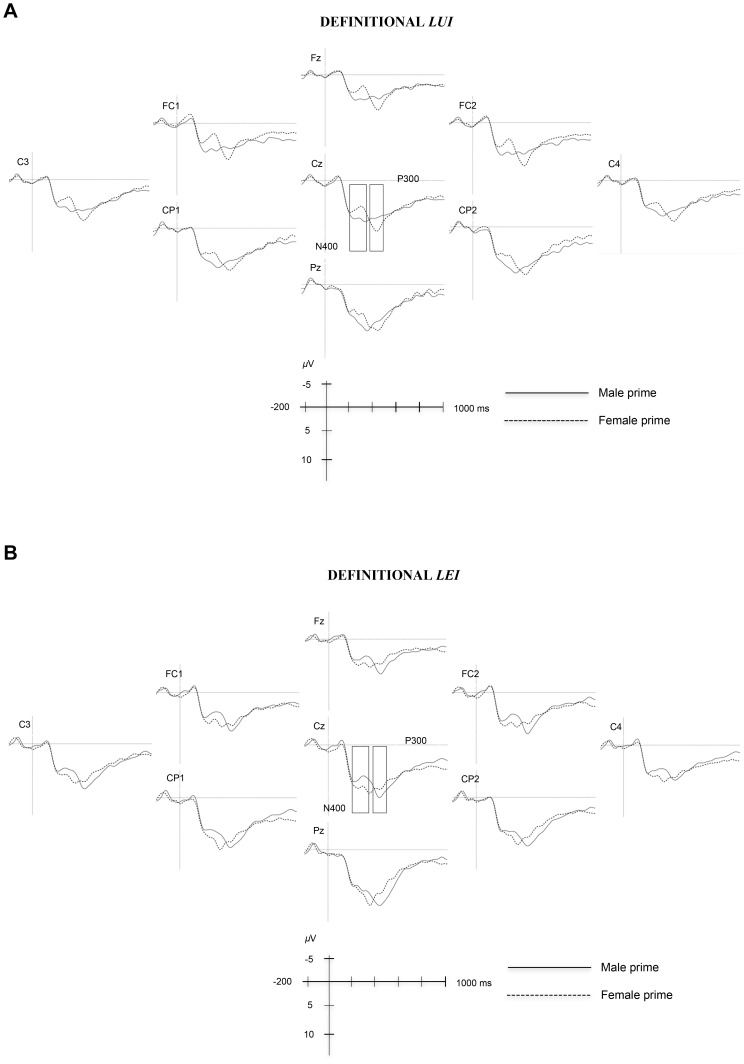
Mean amplitudes evoked by masculine pronouns (lui – “he”) (A) and feminine pronouns (lei – “she”) (B) after male and female definitional primes (n = 25). Zero point is the onset of the pronoun. Amplitudes are provided for representative electrodes only. Negativity is plotted upwards.

**Figure 3:**
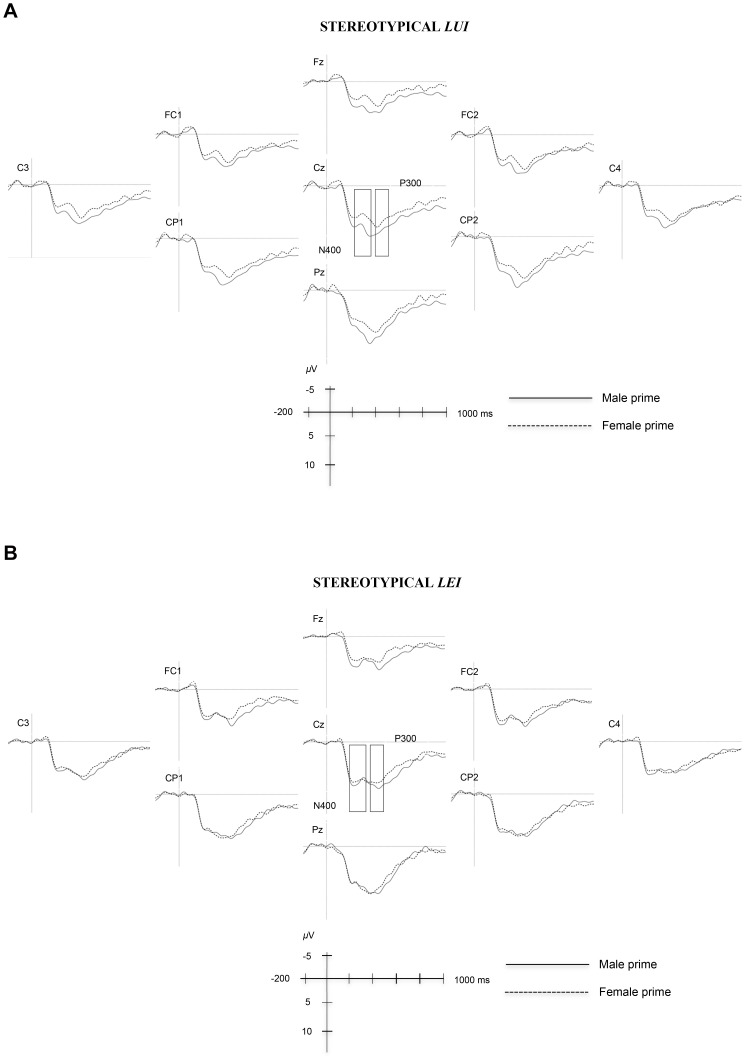
Mean amplitudes evoked by masculine pronouns (lui – “he”) (A) and feminine pronouns (lei – “she”) (B) after male and female stereotypical primes (n = 25). Zero point is the onset of the pronoun. Amplitudes are provided for representative electrodes only. Negativity is plotted upwards.

Figures 2 and 3 plot grand average waveforms in the different experimental conditions. For a clearer inspection of the data, only representative electrodes are plotted. Visual inspection revealed differences in the responses to gender-congruent pronouns in two time windows (200–370 ms and 390–500 ms) following pronoun onset. Statistical analyses on mean amplitude values were carried out in the 200–370 ms and 390–500 ms time windows.

In order to investigate possible topographical differences with respect to the observed effects, mean voltage values at the midline and lateralized electrodes were treated separately. At the midline, omnibus ANOVAs with Electrode (Fz, Cz, Pz, Oz), Prime Type (definitional, stereotypical), Prime Gender (female, male), and Target Gender (feminine, masculine) factors were performed. In addition, separate ANOVAs were run on definitional and stereotypical gender primes. We grouped the remaining electrodes in four regions of five electrodes each for the evaluation of possible topographical differences: Left Anterior (AF3, F7, F3, FC1, FC5), Right Anterior (AF4, F4, F8, FC2, FC6), Left Posterior (CP1, CP5, P3, P7, PO3), and Right Posterior (CP2, CP6, P4, P8, PO4). Repeated measures omnibus ANOVAs were performed on these regions with different levels for Longitude factor (anterior, posterior), Hemisphere factor (left, right), Prime Type (definitional, stereotypical), Prime Gender (female, male), and Target Gender (feminine, masculine). Each of the above four regions of interest was computed such that it contained the *mean* of a group of five electrodes specified above. In addition, separate ANOVAs were run on definitional and stereotypical gender primes. Greenhouse–Geisser correction was applied to *F* values where appropriate.

As the effects under investigation might be influenced by participant gender, a between-subject effect of participant gender (Participant Gender) was included in all ANOVAs conducted. Because the main effects of Participant Gender, Prime Gender, Target Gender, and electrode position, as such, are not central to the question under study, they are not reported. Below, we discuss only those interactions that are of interest given our experimental design and the aims of the study.

#### 200–370 ms window

*Midline.* An omnibus ANOVA on the midline electrodes showed a significant Prime Gender×Target Gender interaction [*F*(1, 23) = 19.9, *p*<.001, **η**_p_^2^ = .46]. Post-hoc tests showed that the priming effect was stronger for masculine pronouns. A significant Electrode×Prime Gender×Target Gender interaction [*F*(3, 69) = 3.4, *p* = .03, **η**_p_^2^ = .13] was obtained. For masculine pronoun, priming effects were present in all four midline electrodes. For feminine pronouns, the priming effect was smaller and mostly concerned central and parietal electrodes.

When separate ANOVAs were run on the two gender types, the gender priming effect was found for definitional primes as evidenced by a significant Prime Gender×Target Gender interaction [*F*(1, 23) = 13.1, *p*<.001, **η**_p_^2^ = .36]. We also observed a significant Electrode×Prime Gender×Target Gender interaction [*F*(3, 69) = 4.9, *p* = .007, **η**_p_^2^ = .18], which showed that the effect of priming was bigger in the central-parietal area. For stereotypical gender, we found a significant Prime Gender×Target Gender interaction [*F*(1, 23) = 7.7, *p* = .01, **η**_p_^2^ = .25]. This interaction showed that the effect of priming was present only for masculine pronouns.

Post-hoc *t*-tests revealed significantly more negative brain responses when participants judged masculine pronouns preceded by incongruent definitional or stereotypical primes (e.g., *lui* preceded by either *passeggera* or *insegnante*) [*F*(1, 23) = 7.5, *p* = .01, **η**_p_^2^ = .25; *F*(1, 23) = 14.9, *p* = .001, **η**_p_^2^ = .39, respectively]. The brain response was also more negative when participants judged feminine pronouns preceded by incongruent definitional primes [*F*(1, 23) = 5.2, *p* = .03, **η**_p_^2^ = .20] (e.g., *lei* preceded by *pensionato*), but, interestingly, not by incongruent stereotypical primes (e.g., *lei* preceded by *conducente*) [*F*(1, 23) = .66, *p* = .42, **η**_p_^2^ = .03]. We will come back to this gender asymmetry effect in General Discussion.

*Lateralized regions.* An omnibus ANOVA on the four lateralized regions showed a significant Prime Gender×Target Gender interaction [*F*(1, 23) = 18.7, *p*<.001, **η**_p_^2^ = .45]. The priming effect was found to be bigger for masculine pronouns than for feminine ones. In addition, we observed a significant Longitude×Prime Gender×Target Gender interaction [*F*(1, 23) = 7.2, *p* = .01, **η**_p_^2^ = .24], with a larger priming effect for masculine pronouns in the posterior region than the anterior one.

When separate ANOVAs were run on the two gender types, the gender priming effect was found for definitional primes as evidenced by a significant Prime Gender×Target Gender interaction [*F*(1, 23) = 16.8, *p*<.001, **η**_p_^2^ = .42]. The ANOVA performed on stereotypical primes showed a significant Longitude×Prime Gender×Target Gender interaction [*F*(1, 23) = 6.8, *p* = .02, **η**_p_^2^ = .23]. The priming effect was observed only for masculine pronouns and was found to be bigger in posterior than anterior region.

Post-hoc *t*-tests on pronouns that followed definitional primes showed that in all four regions (Left Anterior, Right Anterior, Left Posterior, Right Posterior), incongruent pronouns elicited larger negativity than congruent ones for both masculine and feminine pronouns (all *ps*<.05). More negative potentials were obtained when male pronouns were preceded by female than male primes. In line with the significant Longitude×Prime Gender×Target Gender interaction for stereotypical gender, the effect was stronger in posterior than anterior regions [Left Anterior: *t*(24) = 1.8, *p* = .09; Right Anterior: *t*(24) = 2.5, *p* = .02; Left Posterior: *t*(24) = 3.4, *p* = .002; Right Posterior: *t*(24) = 4.3, *p*<.001]. Finally, we observed no significant differences between feminine pronouns after stereotypical congruent or incongruent primes in any regions (all *ps*>.05) (topomaps for this time window can be found in Figure S1, Table S1).

#### 390–500 ms window

*Midline.* An omnibus ANOVA on the midline electrodes showed a significant Prime Gender×Target Gender interaction [*F*(1, 23) = 4.9, *p* = .04, **η**_p_^2^ = .18], as well as significant Prime Type×Prime Gender×Target Gender interaction [*F*(1, 23) = 10.2, *p* = .004, **η**_p_^2^ = .31]. The priming effect occurred only for definitional primes, regardless of the gender of the prime.

To further investigate the three-way interaction (Prime Type×Prime Gender×Target Gender), separate ANOVAs were run on the two gender types. The gender priming effect was found for definitional primes as suggested by a significant Prime Gender×Target Gender interaction [*F*(1, 23) = 10.7, *p* = .003, **η**_p_^2^ = .32] with a priming effect for both masculine and feminine pronouns. No significant interactions were found for stereotypical primes.

Post-hoc *t*-tests revealed significantly more positive brain responses when participants judged masculine and feminine pronouns preceded by incongruent definitional primes than by congruent ones (e.g., *lui* preceded by *passeggera*; *lei* preceded by *pensionato*) [*F*(1, 23) = 4.2, *p* = .05, **η**_p_^2^ = .16; *F*(1, 23) = 8.0, *p* = .01, **η**_p_^2^ = .26, respectively]. For stereotypical gender, the brain response was more negative when participants judged masculine pronouns preceded by incongruent stereotypical primes than by congruent ones [*F*(1, 23) = 6.3, *p* = .02, **η**_p_^2^ = .21] (e.g., *lui* preceded by *insegnante*). It is important to note that this effect goes in the opposite direction with respect to definitional gender. This negativity may suggest that the conflict detected by the brain in the earlier window (see the N400 effect above) was not resolved and the negativity continued into the late window. We will come back to this effect in General Discussion. No differences were observed when feminine pronouns were preceded by stereotypically congruent or incongruent primes [*F*(1, 23) = .2.2, *p* = .15, **η**_p_^2^ = .08] (e.g., *lei* preceded by *conducente* vs. *insegnante*).

*Lateralized regions.* An omnibus ANOVA on the four lateralized regions showed a significant Prime Gender×Target Gender×Participant Gender interaction [*F*(1, 23) = 4.2, *p* = .05, **η**_p_^2^ = .15] with a priming effect only in female participants. We also observed a Prime Type×Prime Gender×Target Gender interaction [*F*(1, 23) = 6.3, *p* = .02, **η**_p_^2^ = .21] with a priming effect only for definitional gender regardless of the gender of the pronoun. In addition, the Hemisphere×Longitude×Prime Type×Prime Gender×Target Gender interaction was found significant [*F*(1, 23) = 7.2, *p* = .01, **η**_p_^2^ = .24].

To further investigate the above interactions, separate ANOVAs were conducted on definitional and stereotypical primes. The ANOVA on definitional primes showed a significant Prime Gender×Target Gender interaction [*F*(1, 23) = 7.7, *p*<.01, **η**_p_^2^ = .25] with a priming effect independent of pronoun gender (i.e., present for masculine, as well as feminine pronouns). Further, we observed a significant Prime Gender×Target Gender×Participant Gender interaction [*F*(1, 23) = 6.0, *p* = .02, **η**_p_^2^ = .20]: again, the priming effect was present *only* in female participants and was independent of the pronoun gender. We will come back to this finding in General Discussion. The ANOVA performed on stereotypical primes did not show any significant interactions.

Post-hoc *t*-tests on definitionally congruent and incongruent masculine pronouns showed significant differences in two regions [Right Anterior *t*(24) = 2.2, *p* = .04, Left Posterior *t*(24) = 2.0, *p* = .05]. Significant differences were observed only in the parietal area [Left Posterior *t*(24) = 2.3, *p* = .03, Right Posterior *t*(24) = 2.1, *p* = .05] for definitionally congruent and incongruent feminine pronouns. These results suggest that definitionally incongruent masculine and feminine pronouns elicited larger positivity than congruent ones. This is in line with the results reported in Midline analyses. *T*-tests on pronouns that followed stereotypical primes showed that comparable potentials were evoked when female pronouns followed incongruent (male) and congruent (female) primes in all four regions (all *ps*>.05). Finally, similar potentials occurred when male pronouns followed incongruent (female) and congruent (male) primes in three out of the four regions (Left Anterior, Right Anterior, and Left Posterior; all *ps*>.05). However, in the Left Posterior region, responses evoked by masculine pronouns after incongruent (feminine) primes were more negative than after congruent (masculine) primes [*t*(24) = 3.1, *p* = .005] (topomaps for this time window can be found in Figure S1, Table S1). This finding goes in the opposite direction with respect to what was observed for definitional primes, but is consistent with the results observed for Midline electrodes. We will come back to this finding in General Discussion.

### Explicit vs. implicit measures of stereotyping

Following Banaji and Hardin [2], we also examined whether participants' explicit gender stereotype beliefs modulated or not the gender priming effect obtained for stereotypical primes. We computed the correlations between the mean scores obtained by each participant in the BSRI and ASI (HS and BS) and two gender priming scores respectively calculated by: (1) subtracting the reaction times (RTs) for the stereotypically congruent conditions from the RTs for the incongruent conditions; (2) subtracting the ERP amplitudes for congruent stereotypical conditions from ERP amplitudes for incongruent stereotypical conditions in both time windows. Because calculating numerous correlations increases the risk of a Type I error, α was set at .01. No significant correlations were found between the scores of the above explicit measures and the gender priming scores obtained via subtractions (Table 2). The lack of significant correlations confirms the dissociation between explicit and implicit measures of gender stereotyping [2], [58].

**Table 2:** Correlations between mean BSRI and ASI (BS and HS) scores and a gender priming score for the stereotypical condition (correlation coefficients/p values)^1^.

	BSRI		ASI HS		ASI BS	
	*lei*	*lui*	*lei*	*lui*	*lei*	*lui*
**RT**	.15/.46	−.11/.58	−.17/.40	.25/.22	−.17/.40	.15/.48
**ERP 200–370 ms**	−.16/.43	.44/.03	.17/.41	.07/.73	.46/.02	−.05/.81
**ERP 390–500 ms**	−.30/.14	.14/.50	.05/.80	.30/.15	.35/.09	.13/.52

1 Because calculating numerous correlations increases the risk of a Type I error, the level of statistical significance of correlation coefficients was adjusted to p<.01.

## General Discussion

We used ERPs to assess the brain response to Italian third-person pronouns (*lui* “he” and *lei* “she”) that were implicitly primed by definitional or stereotypical antecedents (e.g., *passeggera, pensionato*; *inesgnante, conducente*). In line with existing behavioural evidence [2], [4]–[13], our participants were faster to judge the gender of masculine and feminine pronouns when preceded by gender-congruent than gender-incongruent antecedent primes in both the definitional and stereotypical conditions. The behavioural results did not replicate the gender asymmetry previously reported by Cacciari and Padovani [4]: when the gender stereotype conveyed by the prime was female-oriented, they found an inhibition effect in the response to an incongruent pronoun (e.g., *teacher* – *he*). On the contrary, compared to the control condition, no inhibition was found when participants were presented with a masculine role noun followed by an incongruent pronoun (e.g., *engineer* – *she*). This difference may depend at least in part on the presence of a control condition formed by stereotype-neutral role nouns in the Cacciari and Padovani study [4]. Interestingly, in the present study, the gender asymmetry that did not surface at the behavioural level was observed in the brain response.

The ERP results suggest two distinct effects. When the pronouns were preceded by definitionally incongruent primes (e.g., *pensionato*-*lei*; *passeggera*-*lui*), a larger negativity emerged in the 200–370 ms time window peaking around 300 ms. Crucially, a comparable negativity was elicited when masculine pronouns were preceded by stereotypically incongruent primes (e.g., *insegnante-lui*), but not when feminine pronouns were preceded by stereotypically incongruent primes (e.g., *conducente-lei*). In addition, an increased positivity emerged in the 390–500 ms time interval, peaking around 420 ms after stimulus onset, when masculine and feminine pronouns followed definitionally incongruent primes (e.g., *pensionato*-*lei*; *passeggera*-*lui*).

We interpret the negativity observed for definitional and stereotypical gender violations (only for masculine pronouns for the latter) as an N400-like effect. This interpretation is supported by White et al.'s results [22]: they observed larger N400 amplitudes when participants were primed with a gender category (e.g., *men*) stereotypically incongruent with the target word (e.g., *nurturing*). Admittedly, our N400 has an earlier onset than the classic N400 (whose maximal amplitude is typically in the 350–450 ms time window; for an overview see [42]). Our rather early N400 may be due to the fact that the ERPs were measured on short closed-class words (personal pronouns three letters in length). According to Neville et al. [59], the processing of closed-class words is associated with two left anterior negativities: the N280, indexing specific lexical access to the closed-class word, and the N400-700. However, the existence of an electrophysiological marker for the categorical difference between open and closed-class words has been a matter of debate: some ERP studies failed to find evidence of a clear distinction between the two vocabulary classes [60], [61], while other studies suggested that the two negativities are a modulation of the same component shifting in latency on the basis of lexical frequency (see the Lexical Processing Negativity proposed by a number of researchers [62], [63]), and/or word length [64], [65]. Osterhout, Bersick, and McKinnon [64] and Osterhout, Allen, and McLaughlin [65] analyzed closed- versus open-class words and found robust effect of word length on the ERPs. Specifically, pronouns elicited a negativity in the 280–320 ms time window peaking around 300 ms. The authors concluded that open- and closed-class words do not elicit *qualitatively* distinct negativities that index differences in linguistic function (as has been argued by Neville et al. [59]). Rather, different brain responses to open- and closed-class words reflect *quantitatively* distinct (but qualitatively comparable) negativities, which are attributed to high frequency and short length of closed-class words, such as personal pronouns employed in our study. Indeed, the negativity described by Osterhout, Bersick, and McKinnon [64] and Osterhout, Allen, and McLaughlin [65] is comparable to the one observed in this study.

The later positivity peaking at 420 ms for definitional gender violations is in line with the P300 observed in Barber and Carreiras [45]. Barber and Carreiras [45] manipulated grammatical gender using a task that required a binary decision as in the present study. This P300 component is normally observed in priming experiments when an immediate response is required [45], [66]. It is important to note that although the distribution and latency of the P300 is comparable to that of the P600 component (indeed, some researchers have claimed that the P300 and the P600 belong to the same family [67]–[68], but see [69]), there are important differences between the two components. First, as noted by Barber and Carreiras [45], the P600 is a large broad positivity that is maintained for several hundreds of milliseconds (500–900 ms after stimulus onset). Second, this component does not usually have a defined peak. The P300, on the other hand, is a much shorter-lived positivity with a well-defined peak. Figures 2A and 2B show that, in the case of definitional gender, this positivity was maintained for about one hundred milliseconds and had a rather distinct peak around 420 ms. It is further worth noting that although the classic P300 is thought to peak around 300 ms following stimulus onset (hence the name), some studies showed that its peak and latency are often delayed depending on the experimental manipulations [70]. Indeed, Roehm et al. [71] noted that the P300 effect may follow the N400 and thus it may peak later than 300 ms after stimulus presentation. Other studies have also reported late positivity effects at a word level, interpreted as a P300 following a N400 [72]. Crucially for our study, the different ERP correlates for definitional and stereotypical gender violations in the late time window (i.e., the absence of the P300 effect for the violation of stereotypical gender) suggest that stereotypical and definitional gender may reflect different types of knowledge: semantic knowledge for definitional gender with a direct impact on agreement processing similar to grammatical gender [45], and pragmatic, world-knowledge for stereotypical gender [17], [41].

The significant Prime Gender×Target Gender×Participant Gender interaction reported for definitional primes clearly suggested that the effect of priming was present *only* in female participants in the 390–500 ms time window. Electrophysiological differences in processing agreement violations by male and female participants have previously been reported in literature. For example, in Osterhout, Bersick, and McLaughlin [14], female participants exhibited larger positivities in the P600 window than male participants. However, in Osterhout, Bersick, and McLaughlin [14], females had larger positivities than males in both definitional and stereotypical gender violations, while in our study the differences between males and females only concerned definitional primes. Another crucial difference between the two studies is that Osterhout and colleagues [14] found an effect for males in the P600 window (albeit a smaller one compared to females); in contrast, the positivity we observed in the late time window was driven solely by female participants. Some researchers have argued that females are more grammatically aware and competent than males [73] and, hence, they may be more sensitive to agreement violations. It has also been shown that, on average, females are better than males in a wide range of (off-line) language tasks (e.g., verbal fluency, articulation speed, and, in particular, grammar) [74]. Although these findings are in line with ours (and with those of Osterhout, Bersick, & McLaughlin [14]), they are still rather speculative. Clearly, more empirical work is needed to better understand the gender differences observed in the present study.

One more effect observed in the 390–500 ms time window merits attention. The brain response to stereotypically incongruent masculine pronouns was more negative than to stereotypically congruent ones (e.g., *conducente-lui* vs. *insegnante-lui*). As we already noted above, this effect goes in the opposite direction to what was observed for definitional primes in the same time window (i.e., an increased positivity, interpreted as the P300, when masculine and feminine pronouns followed definitionally incongruent primes). A visual inspection of Figure 3A suggests that the negativity observed for stereotypically incongruent masculine pronouns may reflect the earlier negativity (the N400 effect) that persisted into this late window. This may suggest that the conflict associated with the stereotypical gender incongruity (*insegnante-lui*) encountered in the early time window was *not* resolved and continued into the late time window. This is quite different from the very clear effects observed for the violation of definitional gender, where the conflict was first detected and then fully resolved by the time participants responded (Figures 2A and 2B).

Taken together, our ERP results provide further support for on-line effects of stereotypical gender in language comprehension. Remarkably, our ERP results confirmed a previously observed, yet poorly understood, gender stereotype asymmetry [4], [23], in that gender stereotypes affected the brain response to masculine and feminine pronouns differently. Our participants were more accepting of female drivers than male teachers suggesting that gender stereotypes – conveyed by occupation nouns or personal traits – might be more restrictive for females than males. According to social psychologists, one social group (e.g., males) can become more “normative” than another (e.g., females), being the *unmarked normative group* [75]. Miller et al. [76] showed that when asked to think of a prototypical voter, most people think of a male voter exemplar. Researchers have argued that such ‘androcentrism’ is rather common [75], [77]. That is, attitudes, beliefs, and stereotypes are more influenced by male exemplars than female ones [78]. Questions have been raised with regard to how one social group becomes more normative than another. For example, Miller et al. [76] demonstrated that female exemplars were the norm for the category school teachers. One might wonder whether this may depend on the fact that there are more female elementary school teachers than male ones. Interestingly, according to Miller et al. [76], numerical prevalence is not the main factor. In fact, those categories for which men were thought to be prototypical exemplars did *not* necessarily contain more men than women. This may imply that, while *insegnante* (a prototypical female exemplar “teacher”) recruited mostly female category members, *conducente* (a prototypical male exemplar “driver”) recruited both male and female category members. This fits well with our ERP results in that *insegnante-lui* (“teacher-he”) elicited an N400 effect, while *conducente-lei* (“driver-she”) did not. Although our behavioural results showed faster decision times for masculine, as well as feminine congruent pronouns (relative to incongruent ones), the magnitude of the facilitation was smaller for feminine pronouns (*insegnante-lei* vs. *conducente-lei*, 9 ms) than for masculine ones (*conducente-lui* vs. *insegnante-lui*, 15 ms).

Notwithstanding the pervasiveness of gender stereotypes and their significance for theories of social cognition [79]–[81], relatively little is known about the neural correlates underlying their on-line processing. Using a gender priming paradigm and ERPs, we showed that the brain is indeed sensitive to gender-related social stereotypes conveyed by words and to the gender distribution of social roles and traits. As such, the results of the present investigation shed further light on the nature of gender stereotyping within the realm of neuroscience of social cognition.

## Supporting Information

Table S1 **Appendix: A list of stimuli, and their translations, used in the experiment.**
(DOCX)

Figure S1 **Topomaps for feminine (lei – “she”) and masculine (lui – “he”) pronouns in the two critical time windows, created by subtracting congruent definitional and stereotypical conditions from incongruent definitional and stereotypical ones, respectively (n = 25).**
(TIF)
