# Supplementary material for: The Electrophysiological Underpinnings of Processing Gender Stereotypes in Language
Source: PLoS One. 2012 Dec 3;7(12):e48712. doi: 10.1371/journal.pone.0048712 (PMC3513306; doi:10.1371/journal.pone.0048712)
Supplement: Table S1 — Appendix: A list of stimuli, and their translations, used in the experiment. (DOCX) [file pone.0048712.s001.docx]

| **Definitional** | | **Stereotypical** | | **Fillers** |
| --- | --- | --- | --- | --- |
| **masculine** | **feminine** | **masculine** | **feminine** |  |
| ADULTO | NANA | PUGILE | COLF | TUTOR |
| *adult* | *dwarf* | *boxer* | *maid* | *tutor* |
| BIMBO | MUTA | CLOWN | SEXY | OSPITE |
| *baby* | *mute* | *clown* | *sexy* | *guest* |
| SPOSO | AMICA | COACH | DOLCE | PEDANTE |
| *spouse* | *friend* | *coach* | *sweet* | *pedant* |
| VEDOVO | PAGANA | DEEJAY | ESCORT | LICEALE |
| *widower* | *pagan* | *deejay* | *escort* | *high school student* |
| ALUNNO | MATURA | RAPPER | MEDIUM | CONIUGE |
| *pupil* | *mature* | *rapper* | *medium* | *consort* |
| CUGINO | MOROSA | REDUCE | AMANTE | VIVENTE |
| *cousin* | *girlfriend* | *veteran* | *lover* | *living* |
| DILETTO | STUPITA | OREFICE | LOQUACE | PARENTE |
| *beloved* | *astonished* | *goldsmith* | *loquacious* | *relative* |
| COGNATO | ANZIANA | CUSTODE | VERGINE | DOCENTE |
| *brother-in-law* | *old woman* | *attendant* | *virgin* | *teacher* |
| EUROPEO | FIGLIA | GIUDICE | FACILE | PARTNER |
| *European* | *daughter* | *judge* | *easy* | *partner* |
| PURITANO | BISNONNA | FURFANTE | MANICURE | BAGNANTE |
| *puritan* | *great-grandmother* | *scoundrel* | *manicurist* | *bather* |
| NIPOTINO | BUGIARDA | MANOVALE | VEGGENTE | DISABILE |
| *grandson* | *liar* | *labourer* | *future teller* | *disabled* |
| RIPOSATO | FIDANZATA | POMPIERE | AVVENENTE | AIUTANTE |
| *rested* | *fiancée* | *fire-fighter* | *attractive* | *helper* |
| AMMALATO | COMPAGNA | SERGENTE | SENSUALE | PROMOTER |
| *sick* | *partner* | *sergeant* | *sensual* | *promoter* |
| LAUREATO | RAGAZZINA | GANGSTER | SEDUCENTE | CREDENTE |
| *graduated* | *young* *girl* | *gangster* | *seductive* | *believer* |
| STUDIOSO | FORTUNATA | MERCANTE | ATTRAENTE | ADERENTE |
| *studious* | *fortunate* | *merchant* | *attractive* | *supporter* |
| PROMOSSO | NEONATA | MANDANTE | FRAGILE | GENITORE |
| *promoted* | *new-born* | *‘person who sends someone to commit a crime’* | *fragile* | *parent* |
| COSTIPATO | SCOLARA | WEBMASTER | BADANTE | COMPLICE |
| *sick* | *scholar* | *webmaster* | *caregiver* | *accomplice* |
| FIGLIOLO | ASMATICA | PIROMANE | PEDICURE | CANTANTE |
| *son* | *asthmatic* | *pyromaniac* | *pedicurist* | *singer* |
| POVERELLO | CONTATTATA | SOMMELIER | CHIROMANTE | FIGURANTE |
| *poor (person)* | *contacted* | *wine steward* | *palmist* | *walk-on actor* |
| GUARITO | IMPEGNATA | PASTORE | AMOREVOLE | PENDOLARE |
| *recovered* | *busy* | *minister* | *loving* | *commuter* |
| INESPERTO | STRANIERA | FALEGNAME | PETULANTE | OFFERENTE |
| *non-expert* | *foreigner* | *carpenter* | *annoying* | *offerer* |
| PAESANO | ASSONNATA | SKIPPER | PORNOSTAR | INDECENTE |
| *villager* | *sleepy* | *skipper* | *porno start* | *lewd* |
| INQUILINO | ASCOLTATA | CORRIDORE | ECCITANTE | MINORENNE |
| *tenant* | *listened to (person)* | *runner* | *exciting (person)* | *under-age* |
| AFFILIATO | RELIGIOSA | INGEGNERE | SENSIBILE | ASPIRANTE |
| *affiliated* | *religious* | *engineer* | *sensitive* | *aspirant* |
| DOTTORANDO | INDISPOSTA | CONDUCENTE | BABYSITTER | RESIDENTE |
| *PhD student* | *sick* | *driver* | *babysitter* | *resident* |
| PARANOICO | ANTIPATICA | NAVIGANTE | CARTOMANTE | FAMILIARE |
| *paranoiac* | *unpleasant* | *sailor* | *fortune-teller* | *familiar* |
| PENSIONATO | ECCENTRICA | BRACCIANTE | PROVOCANTE | CONOSCENTE |
| *pensioner* | *eccentric* | *labourer* | *provocative* | *acquaintance* |
| INTROVERSO | RICOVERATA | MENDICANTE | CONFIDENTE | INTERINALE |
| *introvert* | *hospitalized* | *beggar* | *confident* | *interim* |
| SVEGLIATO | TRASFERITA | AMBULANTE | DEBUTTANTE | CONVIVENTE |
| *awoken* | *moved* | *street vendor* | *debutante* | *co-habiting* |
| SMEMORATO | PASSEGGERA | INVENTORE | INSEGNANTE | RICEVENTE |
| *forgetful* | *passenger* | *inventor* | *teacher* | *recipient* |
