# Supplementary figures and images for: The Electrophysiological Underpinnings of Processing Gender Stereotypes in Language
Source: PLoS One. 2012 Dec 3;7(12):e48712. doi: 10.1371/journal.pone.0048712 (PMC3513306; doi:10.1371/journal.pone.0048712)

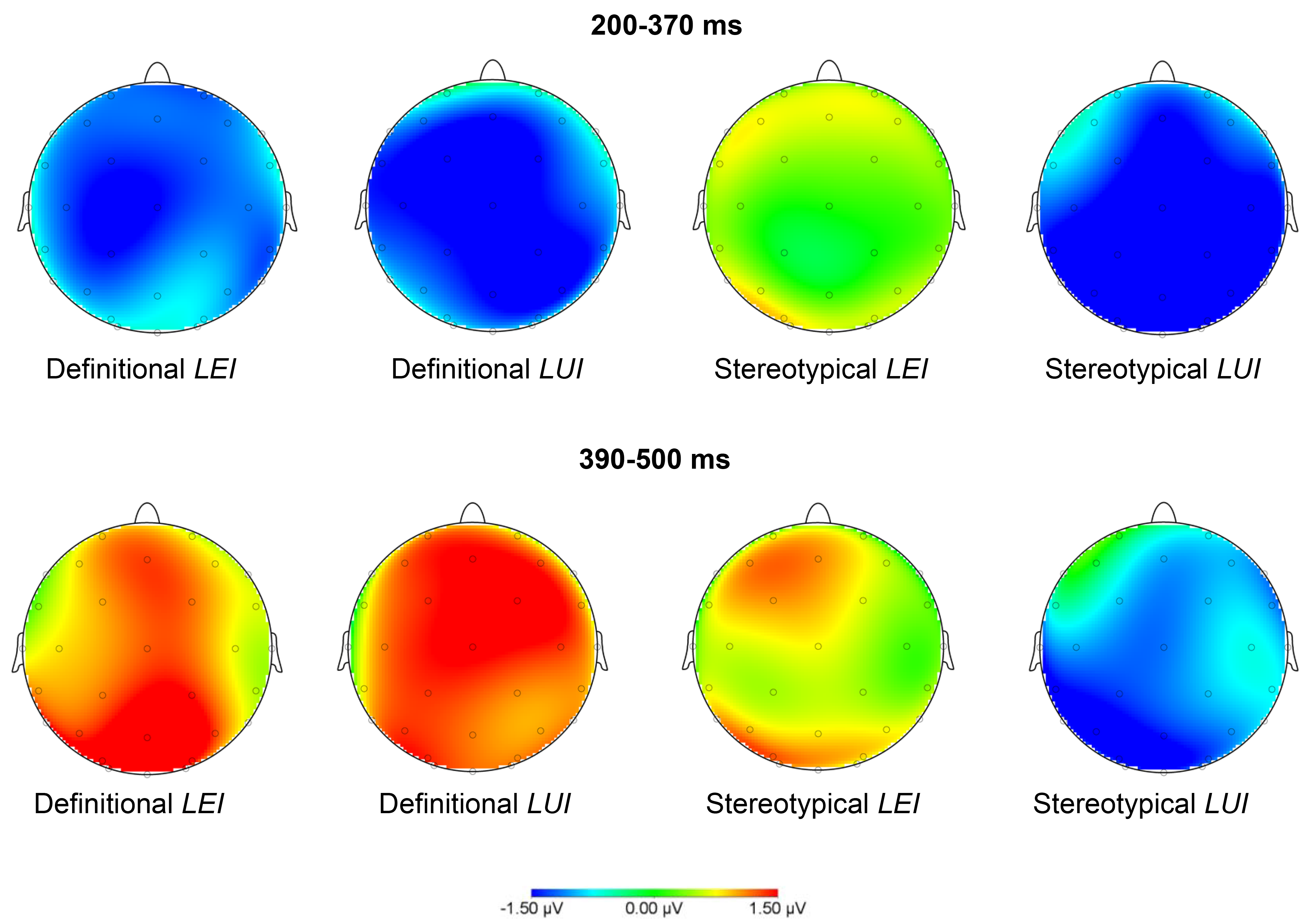

Supplement: Figure S1 — Topomaps for feminine (lei – “she”) and masculine (lui – “he”) pronouns in the two critical time windows, created by subtracting congruent definitional and stereotypical conditions from incongruent definitional and stereotypical ones, respectively (n = 25). (TIF) [file pone.0048712.s002.tif]
